# CRAF R391W is a melanoma driver oncogene

**DOI:** 10.1038/srep27454

**Published:** 2016-06-08

**Authors:** Mohammad Atefi, Bjoern Titz, Jennifer Tsoi, Earl Avramis, Allison Le, Charles Ng, Anastasia Lomova, Amanda Lassen, Michael Friedman, Bartosz Chmielowski, Antoni Ribas, Thomas G. Graeber

**Affiliations:** 1 Department of Medicine, Division of Hematology-Oncology, University of California Los Angeles, Los Angeles, California (UCLA), 90095, USA; 2 Department of Molecular and Medical Pharmacology, UCLA, Los Angeles, CA 90095, USA.; 3 Crump Institute for Molecular Imaging, UCLA, Los Angeles, CA 90095, USA.; 4 New York University, New York, NY 10016, USA.; 5 Jonsson Comprehensive Cancer Center at UCLA, Los Angeles, CA 90095, USA.; 6 Department of Surgery, Division of Surgical-Oncology, UCLA, Los Angeles, CA 90095, USA.; 7 California NanoSystems Institute, UCLA, Los Angeles, CA 90095, USA.

## Abstract

Approximately 75% of melanomas have known driver oncogenic mutations in BRAF, NRAS, GNA11 or GNAQ, while the mutations providing constitutive oncogenic signaling in the remaining melanomas are not known. We established a melanoma cell line from a tumor with none of the common driver mutations. This cell line demonstrated a signaling profile similar to BRAF-mutants, but lacked sensitivity to the BRAF inhibitor vemurafenib. RNA-seq mutation data implicated CRAF R391W as the alternative driver mutation of this melanoma. CRAF R391W was homozygous and over expressed. These melanoma cells were highly sensitive to CRAF, but not BRAF knockdown. In reconstitution experiments, CRAF R391W, but not CRAF WT, transformed NIH3T3 cells in soft-agar colony formation assays, increased kinase activity *in vitro*, induced MAP kinase signaling and conferred vemurafenib resistance. MAP kinase inducing activity was dependent on CRAF dimerization. Thus, CRAF is a bona fide alternative oncogene for BRAF/NRAS/GNAQ/GNA11 wild type melanomas.

The majority of melanomas exhibit elevated activity of the MAP kinase (MAPK) pathway, which is primarily the result of activating mutations in the proteins upstream or within the pathway^1^. The discovery of BRAF mutations in approximately 50% of metastatic melanomas initiated a turning point for this disease with previously very limited treatment options^2^. This led to the development and eventual FDA approval of BRAF and MEK inhibitors^3^,^4^,^5^,^6^,^7^. Occurring in approximately 25% of cases, the second most common driver mutation in melanoma involves the *NRAS* gene^8^. For this molecular subtype of melanoma no strongly effective targeted treatment has been approved and only recently a possible benefit of MEK inhibition has been reported^9^. For the remaining substantial fraction of melanomas the driver oncogenes are still unknown. However, other MAPK pathway activating mutations are probable.

CRAF (RAF1) belongs to the same protein family as BRAF and is positioned at the same level of the MAP kinase signaling cascade. It has been suggested that CRAF is also involved in other activities independent of the MAPK pathway including regulation of effectors such as MST-2 (MAP3K10), ASK-1 (MAP3K5)^10^ and anti-apoptotic factors in mitochondria^11^. In melanoma, CRAF has been mainly implicated in transducing signals downstream of NRAS mutants and as a BRAF dimerization partner in paradoxical signaling^12^ and vemurafenib resistance^13^.

While BRAF was found to be mutated in 8% of all cancers, CRAF demonstrated a significantly lower mutation frequency of 0.7% in cancer cell lines^14^. Although both BRAF and CRAF are expressed and active in melanoma signaling processes, only BRAF shows a high mutation frequency (approximately 50% of melanomas), whereas CRAF mutations are rare and so far have not been demonstrated to generate an alternative activated oncogene. Explanations for this striking difference have implicated the additional levels of negative regulation acting on CRAF, which – unlike BRAF – requires more than one mutation for the activation of an independent high kinase activity with MAPK inducing abilities^14^. Nevertheless, recently, it was found that single amino acid exchanges on CRAF can mediate vemurafenib resistance in BRAF V600E mutant cells^15^. Moreover, even the elevation of CRAF levels has been reported as a potential mechanism of resistance in BRAF mutant melanomas^16^. However, for both resistance mechanisms the *in vivo* occurrence has not been demonstrated.

Here we report the identification of a natural cancer-associated mutation of the *CRAF* gene in both the biopsy of a nodular metastasis from melanoma and its derived cell line, and provide evidence that the identified CRAF R391W mutation causes continuous homodimerization of CRAF, induces high activity of the MAPK pathway and exhibits the characteristics of a bona fide melanoma oncogene.

## Results

### Characterization of the *BRAF* WT/NRAS WT M375 melanoma cell line

**Figure 1:**
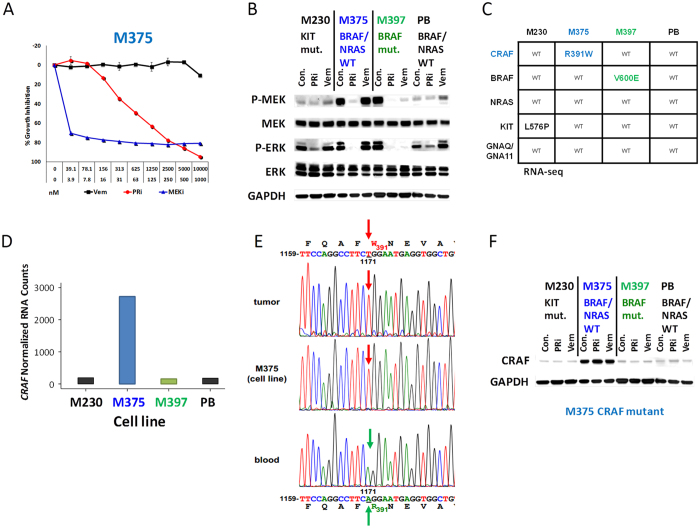
Identification of CRAF R391W as a candidate melanoma oncogene in a BRAF WT/NRAS WT cell line (M375) and matched patient sample. (**A**) Growth inhibition assay demonstrates sensitivity of the BRAF/NRAS wild type cell line M375 to pan-RAF inhibitor (PRi) and MEK inhibitor (MEKi) and its resistance to the BRAF inhibitor vemurafenib. M375 was treated with serial dilutions of these drugs in duplicate. MEKi concentration was 1/10 of the PRi and vemurafenib concentration for each dilution. The assay was repeated twice. Error bars indicate the standard error. (**B**) MAP-kinase signaling and inhibitor treatment response of CRAF mutant M375 cells is reminiscent of BRAF mutated cells except for lack of vemurafenib sensitivity. Western blot assay was performed on CRAF mutant M375, c-KIT mutant M230, BRAF mutant M397 and NRAS/BRAF wild type PB cell lines to analyze their MAPK pathway activities at basal levels or after treatment with 1.0 μM of PRi or vemurafenib for 24 hours. Densitometry results for these blots are shown in Supplementary Figure 2. (**C**) RNA-seq analysis of M375 reveals a homozygous *CRAF* A1171T mutation (CRAF R391W). RNA-seq analysis was performed for the M375, M230 (c-KIT mutant), M397 (BRAF mutant), and PB (BRAF WT/NRAS WT) cell lines. For discovery of the CRAF R391W mutation all mutations detected in the sequencing data were filtered for potentially damaging mutations in the MAP kinase pathway (see methods). The table shows the detected mutations in the common melanoma oncogenes and CRAF for the four cell lines. (**D**) mRNA expression of CRAF in the four cell lines as determined by RNA-seq analysis. M375 shows >10x higher expression of CRAF (R391W). (**E**) *CRAF* A1171T/R391W mutation in M375 and its corresponding tumor is the result of a somatic mutation. Sanger sequencing was performed on the genomic DNA samples isolated from M375 cell line, its corresponding tumor and matched blood of the patient. Sequencing confirmed the presence of *CRAF* A1171T mutation in the tumor, its derived cell line (M375), but not in the blood of the patient. (**F**) Western blot analysis shows strongly elevated abundance of CRAF protein levels in the CRAF mutated melanoma cell line M375 in comparison with 3 other melanoma cell lines.

A 69 year old patient underwent axillary lymph node resection after the finding of stage III melanoma with nodal metastases. One out of seven axillary lymph nodes was involved with melanoma, from which the M375 cell line was established. The M375 cell line was found to be negative for BRAF and NRAS mutations by Sanger sequencing. However, growth inhibition assays suggested that it had constitutively active MAPK signaling, since it was sensitive (IC_50_ = 774 nM) to the pan-RAF inhibitor (pan-RAFi, Amgen Compd A) and to the MEK inhibitor trametinib (IC_50_ < 4 nM). Expectedly, growth of M375 was not inhibited by the BRAF inhibitor vemurafenib, and unlike some other cell lines with wild type BRAF the growth rate of M375 did not paradoxically increase by this drug (Fig. 1A). For comparison, the effect of pan-RAFi and the other inhibitors on the growth of four other melanoma cell lines with different mutations in the MAPK pathway (c-KIT mutant M230, BRAF mutant M397, BRAF WT/NRAS WT PB, and NRAS mutant M311 cell lines), and with a range of sensitivities, are shown in Supplementary Figure 1.

To gauge the activity and pattern of the MAPK pathway response to the inhibitors, M375 and three other melanoma cell lines (M230, M397 and PB), were treated with 1 μM of pan-RAFi, 1 μM of vemurafenib, or solvent alone as the control for 24 h. Interestingly, Western blot analysis indicated that at baseline level, the pattern and levels of MAPK pathway activity (p-MEK and p-ERK) in M375 are more similar to BRAF mutant M397 than the other two tested BRAF WT/NRAS WT cell lines (Fig. 1B, Supplementary Figure 2A,B). The pan-RAFi blocked phosphorylation of p-MEK and p-ERK in both the M375 and M397 cell lines. However, as expected, the BRAF inhibitor vemurafenib was only effective in the BRAF mutant M397 (Fig. 1B). Taken together, the pathway inhibition data implicated the role of an alternative MAPK pathway activator in driving the oncogenic phenotype of M375.

### Identification of CRAF R391W as a candidate melanoma oncogene in the M375 cell line and matched patient samples

Considering the high activity of the MAPK pathway in the BRAF WT/NRAS WT cell line M375 and its sensitivity to pan-RAFi and MEKi, we hypothesized that in this cell line activity of the MAPK pathway is the result of a driving mutation upstream in the MAPK pathway, generating a signaling pattern similar to what is seen with BRAF mutants. To investigate this hypothesis, total RNA from M375 and the three other melanoma cell lines (M230, M397 and PB) was isolated and subjected to RNA-seq analysis (Fig. 1C). This analysis indicated that M375 contains a homozygous A to T mutation at nucleotide 1171 of the *CRAF* gene. This mutation results in an Arg to Trp missense mutation in the CRAF protein (CRAF R391W). To determine CRAF RNA levels, RNA-seq data were analyzed by quantifying read counts of this gene using HTSeq version 0.5.4. and normalizing them by the DESeq R package. Strikingly, RNA expression of CRAF in M375 was at least 10 fold higher than the average CRAF expression in other cell lines (Fig. 1D), which supports a strong positive selection pressure on the mutated allele. To confirm presence of this mutation in the original tumor and to provide evidence that it is the result of a somatic mutation, Sanger sequencing was performed on the genomic DNA samples isolated from M375 cell line, the metastatic lymph node resection, and the matched patient’s blood mononuclear cells. Sequencing confirmed the homozygous *CRAF* A1171T mutation in the cell line and the tumor, but no mutation in the blood sample was found. Therefore, *CRAF* A1171T is a homozygous somatic mutation, which translates to the mutated protein CRAF R391W in the tumor and the cell line (Fig. 1E). Western blot analysis confirmed strongly elevated abundance of CRAF protein levels in the M375 cell line (Fig. 1F). With this, the results strongly implicate mutated CRAF R391W as a candidate alternative driver of oncogenic MAPK signaling.

### Role of CRAF R391W in induction of the MAPK pathway and cellular proliferation

**Figure 2:**
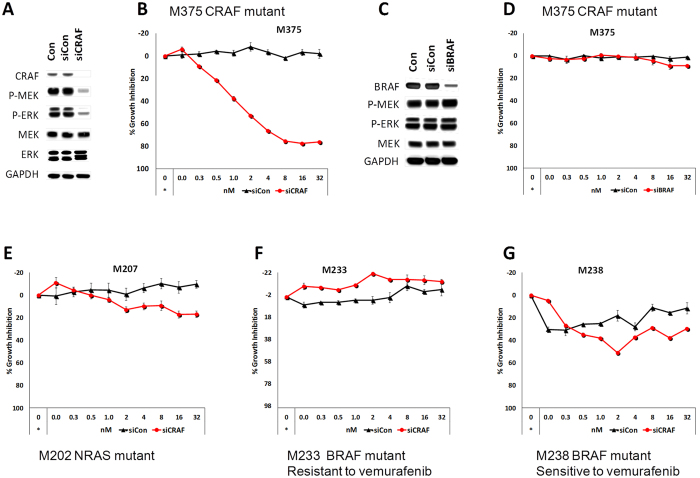
The M375 cell line is dependent on mutated CRAF R391W for MAPK pathway activity and proliferation. (**A**) Knockdown of CRAF inhibits the MAPK pathway in M375. Western blot analysis of the MAPK pathway activity in M375 cell line performed 72 hours after transfection with the non-target (siCon) or CRAF specific siRNA pool. (**B**) Knockdown of CRAF causes a significant growth inhibition in the M375 cell line. The M375 cell line was transfected in a ½ serial dilution with CRAF siRNA or non-target siRNA (siCon) in triplicates. For transfection of siRNAs at all concentrations, a constant amount of Lipofectamine RNAiMAX (0.2%) was used. The data points marked by an asterisk correspond to the control with no siRNA and lipofectamine. (**C**) BRAF knockdown in M375 cells does not affect downstream MAP-kinase signaling. Western blot to analyze the effect of BRAF knockdown on activity of the MAPK pathway activity in the CRAF mutant M375 cell line. The assay was performed 72 hours after transfection with the non-target (siCon) or BRAF specific siRNA pool. (**D**) BRAF knockdown does not inhibit the growth in M375 cells. The assay was performed as describe above. (**E–G**) CRAF knockdown does not cause growth inhibition in a small panel of CRAF wild type, NRAS or BRAF mutant melanoma cell lines. Growth assays performed on the NRAS mutant cell line M207, vemurafenib resistant BRAF mutant cell line M233, and vemurafenib sensitive BRAF mutant cell line M238. Error bars indicate the standard error.

To investigate the importance of CRAF R391W in induction of the MAPK pathway and proliferation of the M375 cell line, expression of this gene was silenced by a CRAF specific siRNA pool. Highly efficient blockade of CRAF expression after 72 hours of siRNA transfection was accompanied by a very substantial down regulation of RAF downstream factors p-MEK and p-ERK (Fig. 2A). Moreover, knockdown of CRAF resulted in a significant growth inhibition of M375 cells. The inhibition of growth was dose-dependent on the amount of CRAF siRNA. Greater than 70% growth inhibition could be achieved by 8 nM of CRAF siRNA, while non-targeted siRNA showed no effect on the growth of the M375 cell line (Fig. 2B). For comparison, the same assays were performed with BRAF siRNA in the M375 cell line, which was verified to significantly down regulate the BRAF protein. However, no decrease in activity of the MAPK pathway or proliferation of this cell line was observed (Fig. 2C,D). Similarly, the growth of this cell line was not affected by the BRAF inhibitor vemurafenib (Fig. 1A). In contrast, CRAF knockdown in a panel of CRAF wild type cell lines (the NRAS mutant cell line M207, the vemurafenib resistant BRAF mutant cell line M233, and the vemurafenib sensitive BRAF mutant cell line M238) had little to no effect on proliferation (Fig. 2E–G). These results indicate that M375 is not strongly dependent on BRAF and that expression of mutated CRAF is critical for activation of the MAPK pathway and proliferation of this cell line.

### CRAF R391W expression reconstitutes MAP-kinase activation dependent on CRAF dimerization

**Figure 3:**
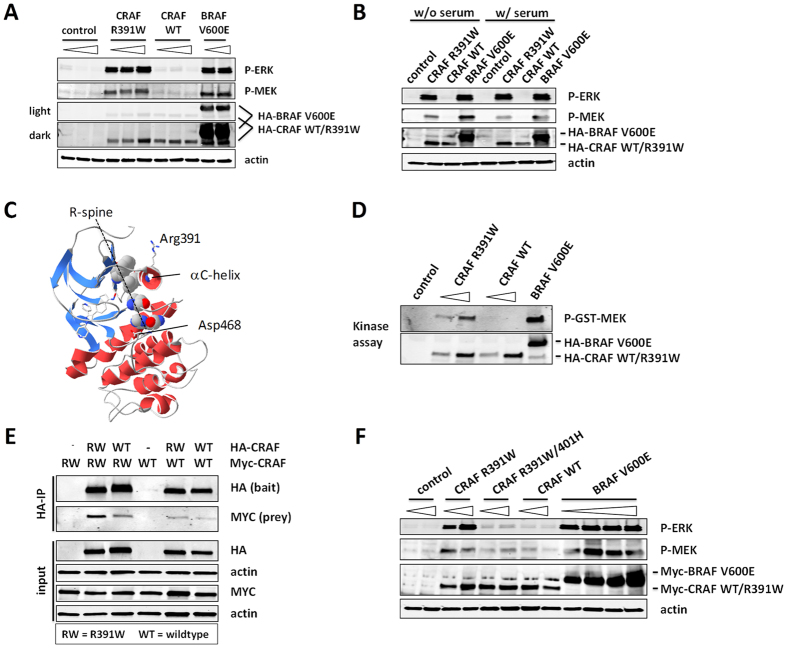
Engineered CRAF R391W expression reconstitutes MAP-kinase activation dependent on CRAF dimerization. (**A**) CRAF R391W activates downstream MAP-kinase signaling. An empty vector control, CRAF R391W, CRAF wild type (WT), and BRAF V600E were transiently expressed in 293T cells and downstream MAP-kinase pathway activation was measured by Western blot after 48 h (p-ERK T202/Y204 and p-MEK S217/221). The N-terminally HA-tagged genes (pDS_HA vector) were transfected in increasing amounts (2, 4, 8 μg DNA per one well of a 6-well plate; 2 and 4 μg for BRAF V600E; as indicated by the gradient triangles). Expression of the transfected genes was tested with an anti-HA antibody. The relatively faint lower band in the HA-BRAF lane, which runs slightly faster than the HA-CRAF protein, appears to be a minor degrative product of the exogenously produced HA-BRAF protein. (**B**) MAP-kinase pathway activation by CRAF R391W does not depend on serum-derived growth factors. Experiment in (**A**) was repeated in the absence (w/o) and presence (w/) of serum. (**C**) Arg391 is located in the αC-helix of CRAF. Structure 3OMV from PDB database visualized with SwissPDB-Viewer^48^,^49^. (**D**) CRAF R391W shows increased kinase activity. The empty control vector (N-terminal HA-tag, pDS_HA, 8 μg DNA per 6cm plate), CRAF R391W (4 and 8 μg DNA), CRAF wild type (WT, 4 and 8 μg DNA), and BRAF V600E (4 μg DNA) were transiently transfected into 293T cells. Anti-HA immunoprecipitation and *in vitro* kinase assay were performed with GST-MEK as the substrate. Phosphorylation of GST-MEK by the precipitated kinases was detected by Western blot. (**E**) CRAF R391W shows increased homo-dimerization. HA- and Myc-tagged constructs of CRAF R391W (RW), CRAF wild type (WT), and the empty HA-vector were transiently transfected into 293T cells. Expression was checked by Western blot (input). The HA-tag was immuno-precipitated and co-precipitation of Myc-tagged proteins was checked by Western blot (HA-IP). (**F**) CRAF R391W activity depends on its dimerization state. The previously described R401H mutation was introduced into CRAF R391W to interfere with functional dimer formation. Downstream MAP-kinase activation of the different constructs was tested as in (**A**) (2 or 4 μg DNA transfected, 0.5, 1, 2, or 4 μg DNA of BRAF V600E).

Next we asked whether the CRAF R391W mutation could induce down-stream MAP kinase pathway activation. For this, we expressed wild type CRAF, CRAF R391W, BRAF V600E, or the empty vector in 293T cells. CRAF R391W strongly induced the MAP-kinase pathway as indicated by activating MEK and ERK phosphorylation (Fig. 3A). The activation level induced by CRAF R391W and BRAF V600E was comparable, whereas CRAF WT had no activating effect. Of note, MAP kinase pathway activation observed for CRAF R391W expression was not dependent on external stimulation by serum components (Fig. 3B).

The R391W mutation is located in the center of the αC-helix of the CRAF kinase domain (Fig. 3C). Movement of the αC-helix is involved in kinase activation^17^,^18^. To test whether MAP kinase pathway activation by CRAF R391W is due to its higher enzymatic activity, we conducted a kinase assay with GST-MEK as the substrate (Fig. 3D). Purified CRAF R391W showed a highly elevated kinase activity compared to CRAF WT. In this experiment, BRAFV600E demonstrated an even higher elevated activity towards phosphorylating MEK. Further experiments would be required to quantitatively determine if BRAFV600E or CRAF R391W has an inherently stronger ability to induce the MAPK pathway.

In most settings activation and signaling of RAF proteins depends on their dimerization state^13^,^19^,^20^. It has been suggested that the αC-helix – with its centrally located R391 residue – is important for RAF protein dimerization^18^,^21^,^22^. We tested combinations of WT and mutant CRAF R391W for homo-dimerization and found that the R391W mutation induces homo-dimerization of CRAF, in comparison to the wild type case. To a lesser but detectable level, the R391W mutation also induces mutant CRAF:wild type CRAF hetero-dimerization (Fig. 3E, Supplementary Figure 3A,B).

To test, whether homo-dimerization is critical for CRAF R391W activity, we tested the effect of the CRAF R401H dimerization mutation. CRAF R401H has been reported to block functional dimerization of CRAF (Supplementary Figure 4)^19^,^20^,^22^. Of note, the R401H mutation does not fully block the physical association of two monomers, but rather is critical for the blocking of dimerization-dependent transactivation of RAF proteins^20^,^22^,^23^. Strikingly, combining R401H with CRAF R391W resulted in a nearly complete loss of down-stream MAP-kinase activation – reverting it back to the substantially reduced activity level of CRAF WT (Fig. 3F). This demonstrates that a single mutation in CRAF (R391W) leads to strongly induced kinase and pathway activity dependent on the formation of functional (trans-activating) dimers.

### CRAF R391W is transforming and can functionally replace BRAF V600E and induce resistance to a BRAF inhibitor

**Figure 4:**
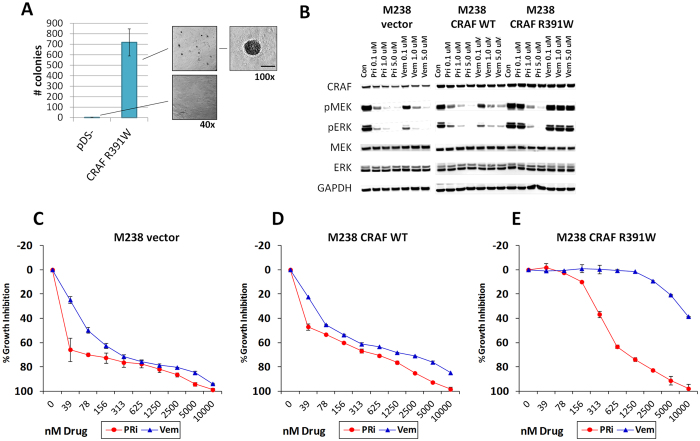
CRAF R391W is transforming and can functionally replace BRAF V600E in BRAFi-treated melanoma cells. (**A**) CRAF R391W induces anchorage independent growth in NIH3T3 cells soft-agar transformation assay. Bar = 250 μm, t-test p-value < 0.05. (**B**) Exogenous expression of CRAF R391W in the vemurafenib sensitive BRAF mutant M238 cell lines abrogates the inhibitory effect of vemurafenib on the MAPK pathway activity. Stable M238 cell lines expressing wild type CRAF (WT), CRAF R391W, or the empty vector (pDS-FB-hyg) were established, treated with increasing concentrations of pan-RAF inhibitor (PRi), vemurafenib (Vem), or the DMSO control for 24 h, and analyzed for downstream MAP-kinase pathway activation by Western blot. (**C–E**) Exogenous expression of CRAF R391W mediates vemurafenib resistance in BRAF mutant M238 cells. Growth inhibition assays performed on the stable M238 cell lines: M238 control (**C**), M238 CRAF WT (**D**) and M238 CRAF R391W (**E**). Cells were treated with serial dilutions of PRi or vemurafenib for 5 days. The assay was performed at least twice. Error bars indicate the standard error.

After confirming the activating effect of CRAF R391W on the signaling level, we asked, whether CRAF R391W expression also induces phenotypic oncogenic alterations. First, we tested whether CRAF R391W is active in a 3T3 soft-agar transformation assay and supports anchorage-independent growth (Fig. 4A). CRAF R391W was transforming in this assay and induced strikingly and significantly more soft-agar colonies than cells carrying the control vector.

Next, we tested, whether exogenous expression of CRAF R391W can functionally replace BRAF V600E in a melanoma cell line. The vemurafenib sensitive BRAF mutant cell line M238 was transduced with an empty retrovector, CRAF WT and CRAF R391W and selected for stable transduction. From each one of these conditions a pool of stably transduced cells was subjected to treatment with 0.1 to 5.0 μM of the BRAFi vemurafenib or the pan-RAFi for 24 hours. Analysis of the MAPK pathway indicated that CRAF R391W could functionally replace BRAF V600E as the CRAF R391W expressing M238 cell line did not show any reduction in p-MEK and p-ERK levels upon treatment with vemurafenib. On the other hand, in the M238 CRAF R391W cell line the MAPK pathway was inhibited by pan-RAFi to a degree similar to the original CRAF mutant cell line M375 (Fig. 1B). Both M238 control and M238 CRAF WT showed inhibition of the MAPK pathway by vemurafenib and pan-RAFi (Fig. 4B).

The growth inhibition response of these three cell lines reflected the response pattern of the MAPK pathway (Fig. 4C–E). Both the M238 control and M238 CRAF WT cell lines were sensitive to vemurafenib and pan-RAFi. This shows their dependence on mutated BRAF as the oncogenic driver and the inability of CRAF WT to compensate for mutated BRAF. On the contrary, the M238 CRAF R391W cell line was substantially resistant to vemurafenib, but still demonstrated growth inhibition by pan-RAFi. Upon treatment with these inhibitors, cell cycle analysis of M238 engineered cell lines showed patterns consistent with cell cycle arrest being a substantial component of the observed growth inhibition results (Supplementary Figure 5A). Measuring cleaved PARP also indicated that exogenous expression of CRAF R391W prevented induction of apoptosis by vemurafenib in M238 CRAF R391W cells while these cells showed significant apoptosis upon treatment with pan-RAFi and MEKi (Supplementary Figure 5B). These results demonstrate that in a melanoma cell line context, CRAF R391W can assume an oncogenic driver role and compensate for the loss of the oncogenic function of mutated BRAF.

### The previously described CRAF E478K mutation is less activating and requires an upstream mutation for full activity

After establishing CRAF R391W as a driver oncogene for the M375 cell line and original tumor, we asked how this mutation compares to CRAF E478K. CRAF E478K has been previously identified as an activating and potential oncogenic mutation for melanoma^8^ and colorectal carcinoma^14^.

**Figure 5:**
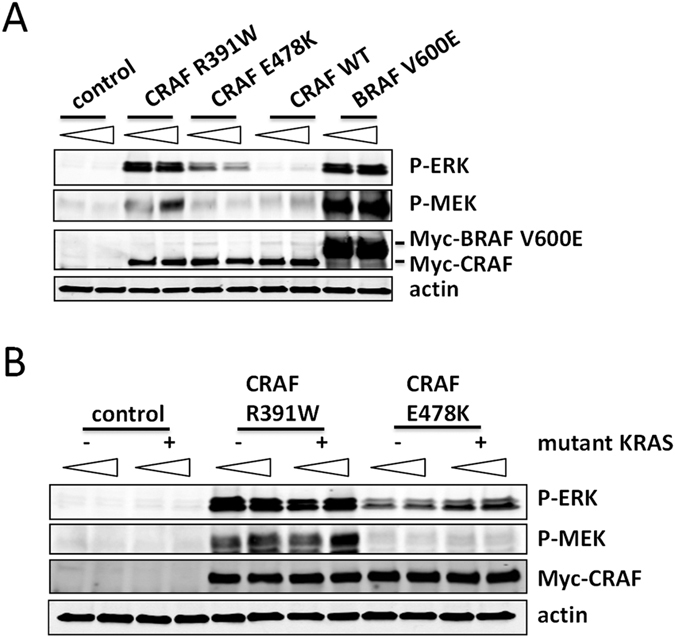
Higher endogenous activity of CRAF R391W than the previously described less active and upstream mutation-dependent CRAF E478K. (**A**) CRAF E478K activates downstream MAP-kinase signaling to a lesser extent than CRAF R391W. An empty vector control, CRAF R391W, CRAF E478K, CRAF wild type (WT), and BRAF V600E were transiently expressed in 293T cells and downstream MAP-kinase pathway activation was measured by Western blot after 48 h (p-ERK T202/Y204 and p-MEK S217/221). The N-terminally Myc-tagged genes (pDS_Myc vector) were transfected in two amounts (2 and 4 μg DNA per one well of 6-well plate; triangles). Expression of the transfected genes was tested with an anti-Myc antibody. (**B**) Co-expression of CRAF E478K and mutant KRAS V12 enhances its activation of downstream MAP-kinase signaling. The control and CRAF mutant plasmids (pDS_Myc) were co-transfected with KRAS V12 or its empty vector (pDS-neo) in two different amounts: 1 μg or 2 μg of each plasmid. Downstream MAP-kinase pathway activation was measured by Western blot after 48 h as above.

We expressed CRAF R391W, CRAF E478K, and controls in 293T cells and analyzed downstream MAP kinase activation by Western blot (Fig. 5A). While CRAF E478K showed increased activation compared to CRAF WT, its inducing effect was notably lower than the level observed for CRAF R391W. This is in agreement with previous reports, which concluded that CRAF E478K acts as an oncogene in conjunction with other activating mutations such as an upstream RAS mutation^14^. We were able to confirm that CRAF E478K can work in conjunction with a KRAS mutant (V12) to activate the MAP kinase pathway, whereas CRAF R391W without oncogenic RAS already induces strong pathway activity (Fig. 5B). While we observed that MAP kinase pathway activation by CRAF R391W can occur in the absence of serum (Fig. 3B), it remains to be determined if participation of wildtype RAS activation is required, or if, as is the case for BRAFV600E^24^, signaling activation by CRAF R391W is completely independent of upstream RAS involvement. In sum, CRAF R391W represents an oncogenic mutation in melanoma that independent of oncogenic RAS can drive a high level of downstream MAP-kinase pathway activation and support oncogenic transformation.

## Discussion

In this study, we identified a CRAF R391W missense mutation in a melanoma metastasis biopsy and its derived cell line. This CRAF mutation shows the critical aspects of an oncogenic driver mutation: homozygosity and highly amplified expression of this locus indicates strong positive selection pressure^25^; the derived melanoma cell line is strictly dependent on CRAF (R391W) expression for activity of MAPK signaling and proliferation; exogenous CRAF R391W expression strongly enhances kinase activity, homodimerization and induces downstream MAP kinase signaling; and CRAF R391W induces anchorage independent growth and can functionally replace BRAF V600E in a melanoma cell line.

The kinase family members CRAF and BRAF both activate MAP kinase signaling and are considered oncogenes^2^,^26^. However, CRAF mutations are much less frequent in human cancers. For example, a study found BRAF mutations in 8% of cancer cell lines, whereas CRAF was only mutated in 0.7% of the tested cell lines^14^. In a compendium of datasets from recent cancer sequencing projects, 0.6% of samples showed a CRAF mutation (up to 4% for a single cancer type) and 4.3% a BRAF mutation (up to 58% for a single cancer type) (as of Nov. 2013)^27^. The TCGA project with 228 melanoma samples detected BRAF mutations in 51% and CRAF mutations in 4% of the melanoma cases. Upon our search, two samples in this database showed a CRAF mutation at position R391: one melanoma with R391S mutation (TCGA-EE-A20C, concurrent with a NRAS Q61R mutation) and one renal papillary cell carcinoma with R391W mutation (TCGA-B3-3925, without RAS mutation). In addition, the corresponding amino acid in BRAF (K499E/N) has been found mutated in the congenital cardiofaciocutaneous syndrome 1 (CFC1) disorder^28^,^29^,^30^. Strikingly, this syndrome is associated with MAP kinase pathway dysregulation and BRAF K499E has been shown to activate the pathway^28^,^29^.

Several studies have confirmed that single amino acid substitutions can activate CRAF, convert CRAF into a transforming oncogenic kinase, and mediate BRAF inhibitor resistance^14^,^15^,^31^,^32^,^33^,^34^. Our study extends this work by demonstrating that mutated CRAF can be a potent alternative oncogene for malignant melanoma. The question arises why BRAF mutations are much more frequent than CRAF mutations. Previously, it has been suggested that tighter regulation of CRAF by its inhibitory N-region suppresses activity of CRAF mutations below their transformation threshold^14^. In the prior work, CRAF E478K was presented as an example for a mutation that is not sufficiently active as an oncogene due to inhibitory regulation of the N-region. In our comparison, CRAF R391W shows substantially stronger induction of the MAP kinase pathway than CRAF E478K (Fig. 5), and demonstrates an activating effect comparable to BRAF V600E (Fig. 3). Thus, mutant CRAF can act as a potent oncogene and alternative explanations need to be examined for the reduced observed mutational occurrence of CRAF compared to BRAF. For example, it is possible that the mutation rates for BRAF and CRAF are different or CRAF is more constrained by its regulatory network^14^. In addition, the possibility remains that some of the previous studies have missed oncogenic genetic events that might activate CRAF through structural variations of the inhibitory N-terminus due to the employed sequencing and analysis approaches^26^,^35^,^36^.

How does the R391W mutation activate CRAF and downstream MAP kinase signaling? Arg-391 is centrally located in the αC-helix of the kinase domain of CRAF (Fig. 3C). Since movement of the αC-helix is involved in kinase activation^17^,^18^, this mutation could directly stabilize the active kinase conformation. However, it has also been suggested that the αC-helix is important for RAF dimerization^18^,^21^,^22^, which in most settings is crucial for its activity^13^,^19^,^20^. We found that both CRAF kinase activity and homo-dimerization are induced by R391W and – unlike BRAF V600E^37^ – this activity depends on functional dimerization (Fig. 3).

Melanoma has been characterized as a cancer that is mainly driven by MAP kinase pathway dysregulation^1^. At the onset of our study we noticed the striking similarity of the signaling pattern of the M375 cell line and BRAF V600E cell lines (Fig. 1). This led to the discovery of CRAF R391W as an alternative oncogene that drives the MAP kinase pathway. This finding further underscores the importance of MAP kinase signaling for melanoma. Importantly, this finding suggests that MAP kinase pathway inhibition can – in principle –constitute a viable treatment option for a subset of BRAF/NRAS wild type melanomas. For example, whereas the M375 cell line is resistant to BRAF inhibition by vemurafenib, it is sensitive both to pan-RAF and MEK inhibition (Fig. 1).

In conclusion, we have discovered and characterized CRAF R391W as a novel oncogene for BRAF/NRAS wild type melanomas. This is the first report of CRAF as a bona fide oncogene in melanoma and adds to the reports of oncogenic CRAF variants in other cancers^36^.

## Materials and Methods

### Reagents and Cell Lines

Vemurafenib and trametinib were purchased from Selleck Chemicals (Houston, TX) and the pan-RAF inhibitor (pan-RAFi, Amgen Compd A^38^) was obtained from Amgen (Thousand Oaks, CA) under a materials transfer agreement (MTA). Human melanoma cell lines used in this study were established from patient’s biopsies under UCLA IRB approval #11-003254. Maintenance and mycoplasma testing of the cell lines were performed as it was described before^39^.

### RNA-seq analysis & DNA sequencing

Presence of mutations in the genes of interest were investigated by Sanger sequencing, OncoMap 3, Iontrone^39^,^40^ or RNA-seq. For RNA-seq, cells were harvested and subjected to RNA isolation by RNeasy kit (Qiagen, Valencia, CA) according to the manufacturer instruction. Total RNA samples were converted into cDNA libraries using a modification to the Illumina TruSeq RNA Sample Preparation Kit v2 to impart strand specificity. cDNA was then amplified by PCR. Final cDNA libraries were analyzed for size distribution using an Agilent Bioanalyzer, quantitated by qPCR and normalized to a concentration of 2 nM prior to submission to Expression Analysis, Inc. (Durham, NC) for 50 bp paired end sequencing on the Illumina platform. Read mapping was performed using TopHat version 2.0.9 with the NCBI Build 37 reference genome^41^. Read counts per gene were quantified using HTSeq version 0.5.4 and normalized using the DESeq R package^42^. Variant calling was performed using the Genome Analysis Toolkit version 2.7 and annotated using Annovar^43^,^44^. To analyze the *CRAF* mutation, DNA was isolated from the patient tumor sample, the patient’s blood, and the patient-derived melanoma cell line M375. For this, the “DNeasy Blood and Tissue Kit” (Qiagen, Valencia, CA) was used. The patient FFPE tumor sample was subjected to xylene/ethanol treatment before DNA isolation. The relevant region of the *CRAF* gene was amplified with the Phusion High-Fidelity DNA Polymerase (NEB, Ipswich, MA) (forward primer: CTA ACT GTA GCG ATA GCA GTT AAC C; reverse primer: GCA CAG TCC ACT AAC TCT ACA GTC) and submitted for sequencing at the UCLA sequencing core facility (sequencing primer: TGGATAAATGATTCACTGTATCTTCC).

### Growth inhibition and transformation assays

Pharmacological growth inhibition assays were performed as previously described^39^. Each condition was in duplicate and assays were repeated at least twice. For siRNA experiments, knockdown of the target genes by siRNA was performed as it was described before^39^. In brief, cell lines were transfected with 32 nM of specific siRNA for either CRAF or BRAF or no target siRNAs (Dharmacon, Lafayette, CO) by using 0.2% of Lipofectamine^®^ RNAiMAX (Life technologies, Carlsbad, CA). Knockdown growth inhibition assays were performed by transfecting the cell lines with the ½ serial dilutions of the target siRNA or non-targets siRNA (siCon) in triplicates. The highest concentration of each siRNA was 32 nM. For transfection of siRNAs at all concentrations, a constant amount of Lipofectamine^®^ RNAiMAX (0.2%) was used.

NIH3T3 cells stably transfected with CRAF R391W or the empty retroviral vector (pDS-FB-hyg) and were plated in soft-agar plates. A 0.8% (w/v) agarose base layer with 0.5x DMEM + 10% FBS + antibiotics media was prepared in 6cm plates. 1 × 10^5^ cells were plated on the base layer in 0.35% agarose and 0.75x DMEM + 10% FBS + antibiotics. The assay was performed in triplicates. The plates were supplemented with fresh media every 3–5 days. Colonies were counted and microscopic images taken after 24 days.

### Inhibitor treatment and Western Blot experiments

Western blotting was performed as previously described^39^. The conditions and duration of treatments of the cells before protein isolation are described in the text and the figure legends. Primary antibodies included p-ERK Thr204/205, ERK, p-MEK Ser217/221, MEK, anti-HA (clone C29F4) and GAPDH (all from Cell Signaling Technology, Danvers, MA), BRAF (H-145) and CRAF (E-10) (both from Santa Cruz biotechnology, Santa Cruz, CA), anti-MYC and anti-actin (Sigma Aldrich, St. Louis, MO). Secondary antibodies were HRP conjugates (Santa Cruz biotechnology, Santa Cruz, CA). Densitometry of bands was performed by using ImageJ program.

### Cell Cycle Analysis and Apoptosis

Cell lines were treated with DMSO, 1.0 μM of vemurafenib, 1.0 μM of pan-RAFi, or 25 nM of MEKi for 48 hours before the harvest, fixation and DAPI staining for cell cycle analysis^45^. For apoptosis assay, all treatments and preparations were similar to the cell cycle analysis. Cells were stained for cleaved poly [ADP-ribose] polymerase (PARP) by anti–PARP-Alexafluor700 (clone F21–852; BD Biosciences). LSRII (BD Biosciences) flow cytometry machine was used for both cell cycle analysis and cleaved PARP positive cell detection.

### CRAF/BRAF expression constructs

A CRAF plasmid (pDONR223-CRAF), KRAS V12 plasmid (pDONR223-K-RAS V12), and BRAF V600E plasmid (pBabe-Puro-BRAF-V600E) were obtained from Addgene (Cambridge, MA). BRAF V600E was PCR amplified and cloned into a Gateway-system entry plasmid (pDonr207) (life, Carlsbad, CA). CRAF was transferred into the pDonr207 plasmid and a stop codon was added by a similar procedure. The following CRAF mutations were generated by overlap extension PCR and re-cloning into the pDonr207 plasmid by BP recombination: CRAF R391W, CRAF R391W R401H, and CRAF E478K. These entry clones were transferred into the following destination vectors: pDS_MYC and pDS_HA (n-terminal epitope tags; Alliance for Cellular Signaling), pDS-FB-hyg (retroviral expression vector, Alliance for Cellular Signaling), and pCEMM-CTAP-GWY (retroviral expression vector; GFP marker; tag not expressed due to presence of stop codon)^46^. Retroviral particles were produced as described previously^47^. Virus supernatants were harvested after 48 h and filtered. For the transduction, the cells were incubated with the viral supernatant and 4 μg/ml polybrene.

### MAP-kinase pathway activation by CRAF mutants

For the analysis of downstream MAP-kinase pathway activation by CRAF/BRAF constructs, 293T cells were transfected with the expression plasmids using Lipofectamine 2000 (Life, Carlsbad CA). Cells were cultured in DMEM + 10% FBS + antibiotics except when explicitly stated that serum (FBS) was omitted. Forty eight hours after transfection the cells were lysed with mRIPA buffer (150 mM NaCl, 1% (v/v) NP-40, 0.25% (w/v) Na-deoxycholate, 1 mM EDTA, 50 mM Tris/HCl, pH = 7.4) with proteinase (1 mM PMSF, 20 μg/ml leupeptin, 20 μg/ml aprotinin) and phosphatase inhibitors (10 mM NaF, 10 mM beta-glycerophosphate, 10 mM Na-pyrophosphate, and 1 mM Na_3_VO_4_). Western blots were performed using a standard protocol, infrared secondary antibodies were used (IRDye 680 goat anti-rabbit and IRDye 800CW goat anti-mouse, LI-COR, Lincoln, NE), and the blots scanned with a LI-COR scanner (LI-COR Lincoln, NE).

### Kinase activity assay

293T cells were transfected with the empty HA-tag expression plasmid (pDS_HA vector) or HA-tagged constructs of CRAF R391W, CRAF wild type, and BRAF V600E using Lipofectamine 2000 (Life, Carlsbad, CA). Forty eight hours after transfection the cells were lysed in lysis buffer (125 mM NaCl, 0.2% (v/v) NP-40, 50 mM Tris/HCl, pH = 7.5, 1.5 mM MgCl_2_, 5% (v/v) glycerol and proteinase and phosphatase inhibitors) with two freeze-thaw cycles in N_2_(l). The HA-tagged proteins were immunoprecipitated with anti-HA magnetic beads (Pierce, Thermo Fisher, Rockford, IL) for 2 h at 4 °C. The immunoprecipitates were washed thrice with lysis buffer. The *in vitro* kinase assay was conducted using the B-Raf Assay Kit (EMD Millipore, Billerica, MA). Briefly, 20 ul Mg/ATP cocktail, 1 μg GST-MEK1 (inactive), and 22 ul ADBI buffer were added to the beads and incubated for 30 min at 30 °C. The samples were boiled in SDS sample buffer and analyzed by LI-COR Western blot as described above.

### Co-immunoprecipitation

293T cells were co-transfected with pairs of HA- and MYC-tagged constructs (pDS_HA and pDS_MYC vector) of CRAF wild type, CRAF R391W, or CRAF R391W R401H (or the empty vector) by using Lipofectamine 2000 (Life, Carlsbad, CA). Forty eight hours after transfection the cells were lysed in lysis buffer (as described in kinase assay). The HA-tagged proteins were immunoprecipitated with anti-HA magnetic beads (Pierce, Thermo Fisher, Rockford, IL) overnight at 4 °C. The immunoprecipitates were washed thrice with lysis buffer. Co-immunoprecipitates were eluted with 0.1 M glycine, pH = 2.0 and neutralized with 1 M Tris/HCl, pH = 8.5. The input fractions and elutes were analyzed by Western Blot as described above.

## Additional Information

**How to cite this article**: Atefi, M. *et al.* CRAF R391W is a melanoma driver oncogene. *Sci. Rep.* **6**, 27454; doi: 10.1038/srep27454 (2016).

## Supplementary Material

Supplementary Information
