# Supplementary material for: CRAF R391W is a melanoma driver oncogene
Source: Sci Rep. 2016 Jun 8;6:27454. doi: 10.1038/srep27454 (PMC4897636; doi:10.1038/srep27454)
Supplement: Supplementary Information [file srep27454-s1.pdf]

CRAF R391W is a melanoma driver oncogene

Mohammad Atefi*, Bjoern Titz*, Jennifer Tsoi, Earl Avramis, Allison Le, Charles Ng, Anastasia Lomova, Amanda Lassen, Michael Friedman, Bartosz Chmielowski, Antoni Ribas and Thomas G. Graeber

* Contributed equally

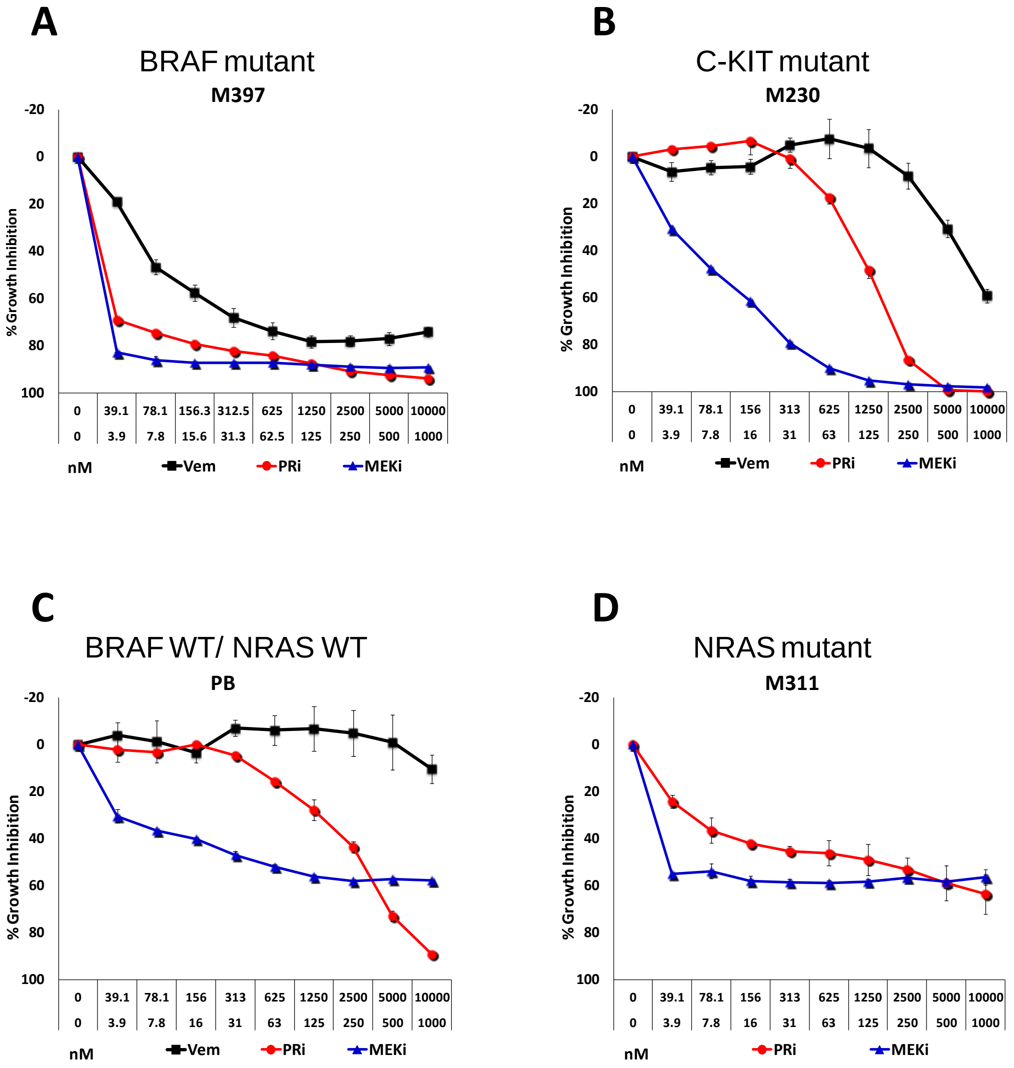

Supplementary Figure 1. Dependence on mutant RAF causes higher sensitivities to the inhibitors of MAPK pathway. (A) BRAF V600E mutant cell line M397 is highly sensitive to the BRAF inhibitor vemurafenib, pan-RAFi (PRi) and MEKi. (B) Resistance of c-KIT mutant M230 melanoma cell line to vemurafenib and partial resistance to PRi and MEKi. (C) Resistance of NRAS/BRAF wild type melanoma cell line to all three MAPK pathway inhibitors. Cell lines were treated with serial dilutions of these drugs in duplicate. MEKi concentration was 1/10 of the PRi and vemurafenib for each dilution. The assays were repeated twice. (D) M311 is a NRAS mutant cell line relatively resistant to pan-RAFi and MEKi. Error bars indicate the standard error.

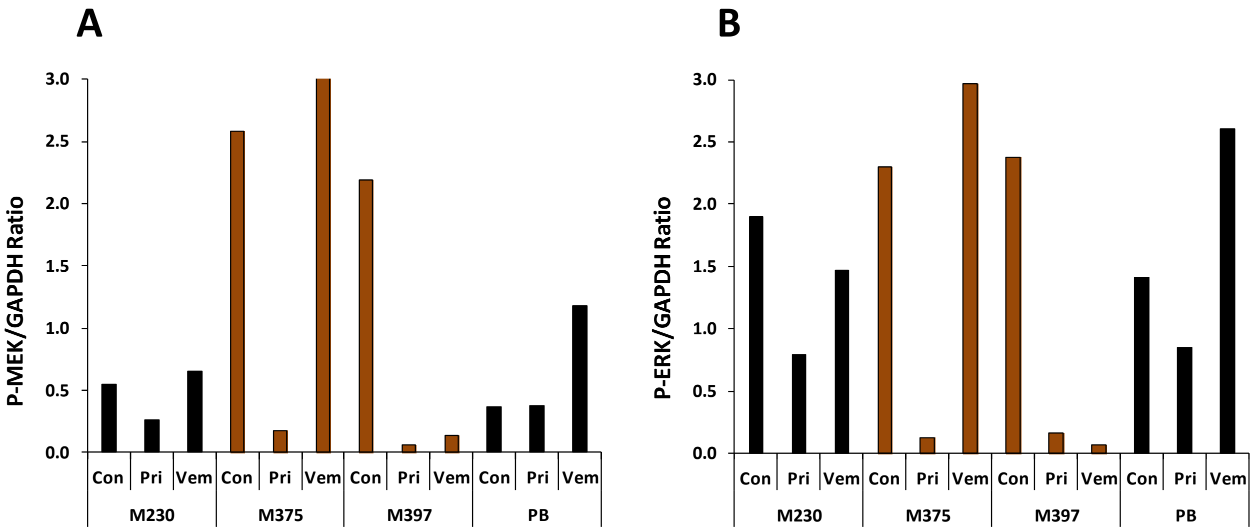

Supplementary Figure 2. Densitometry of Western blot assays that were performed on CRAF mutant M375, c-KIT mutant M230, BRAF mutant M397 and NRAS/BRAF wild type PB cell lines to analyze their MAPK pathway activities at basal levels or after treatment with 1.0µM of PRi or vemurafenib for 24 hours. The pattern of MAP-kinase pathway activity in the CRAF mutant M375 cells shows similarity with the pattern of this pathway activity in the BRAF mutated cell line M397, except for the lack of vemurafenib sensitivity.

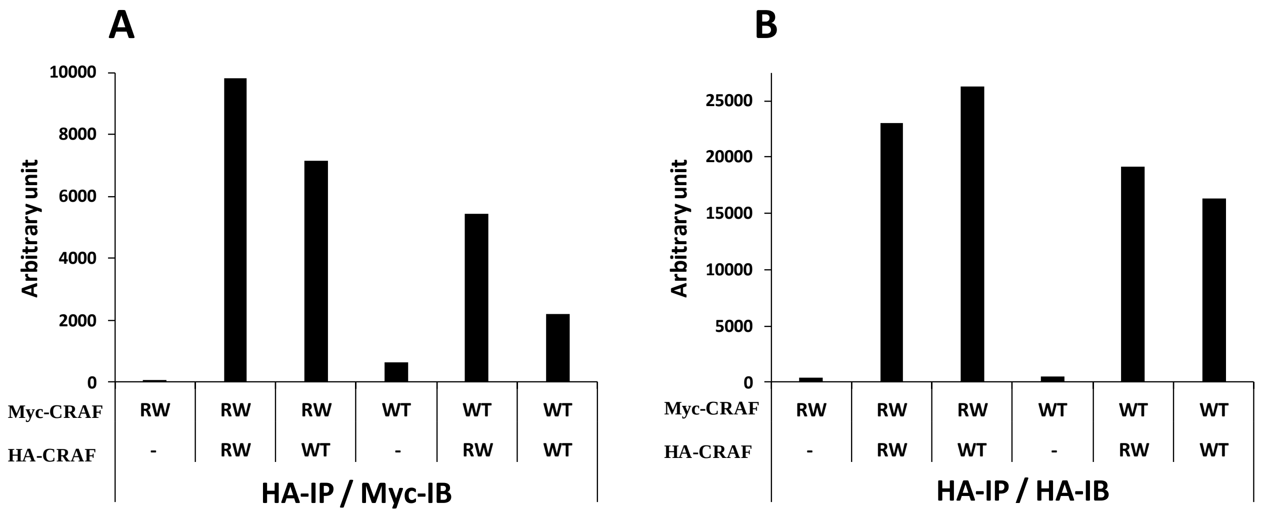

Supplementary Figure 3. Densitometry of immuno-precipitation assays indicating higher levels of homo-dimerization of CRAF R391W and also dimerization of CRAFR391W and CRAFWT. HA- and Myc-tagged constructs of CRAF R391W (RW), CRAF wild type (WT), and the empty HA-vector were transiently transfected into 293T cells. The co-precipitation of Myc-tagged proteins with the HA-tag immuno-precipitated (IP) proteins was checked by Western blot (Myc-immuno blot (IB) in panel A, control HA-IB in panel B).

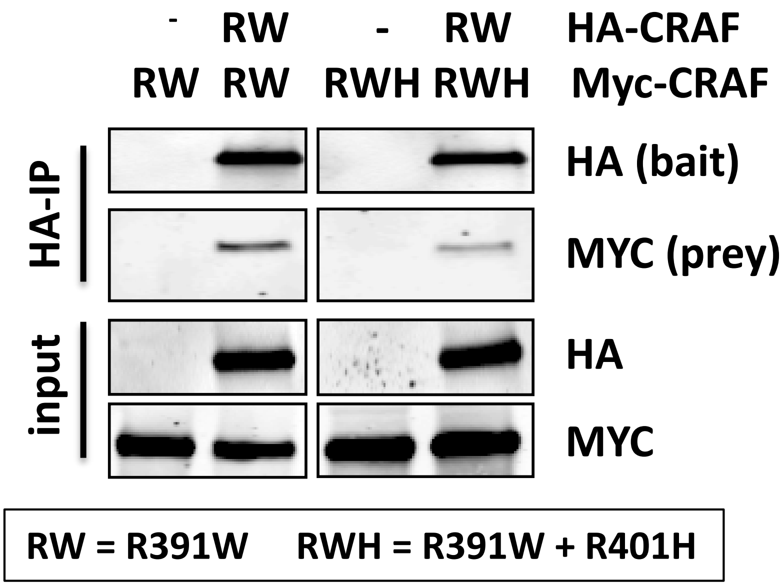

Supplementary Figure 4. CRAF R401H mutation interferes with CRAF R391W homo-dimerization. HA- and Myc-tagged constructs of CRAF R391W (RW), CRAF R391W R401H (RWH), and the empty HA-vector were transiently transfected into 293T cells. Expression was checked by Western blot (input). The HA-tag was immuno-precipitated (HA-IP) and co-precipitation of Myc-tagged proteins was checked by Western blot.

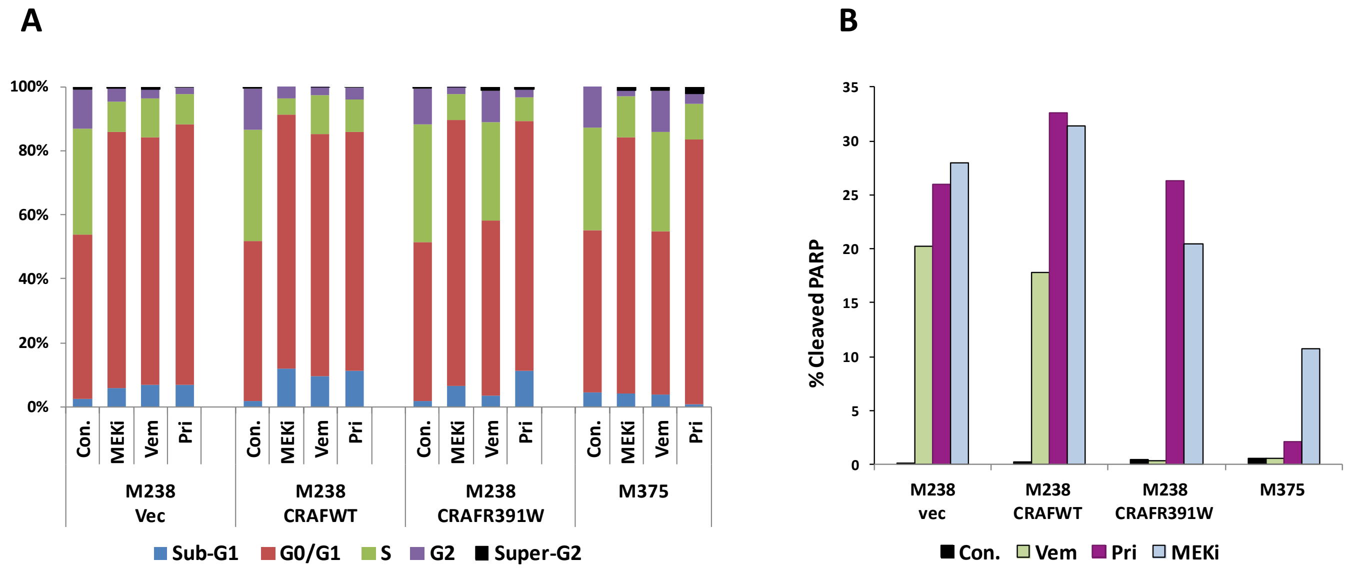

Supplementary Figure 5. CRAF R391W can functionally replace BRAF V600E and causes resistance to vemurafenib indicated by cell cycle analysis and apoptosis assay. (A) Expression of CRAF R391W causes resistance to vemurafenib while pan-RAFi (PRi) and MEKi still can block the cell cycle progression. The empty vector (pDS-FB-hyg), CRAF WT or CRAF R391W genes were stably expressed in BRAF mutant vemurafenib sensitive cell line. Transduced M238 sub cell lines and M375 were treated with 1µM of vemurafenib, 1µM of PRi or 25nM of MEKi and after 48h were fixed and stained with DAPI and analyzed for cell cycle analysis. (B) Lack of apoptotic response to vemurafenib in cell lines with CRAF R391W expression. Apoptotic response was detected by staining for cleaved PARP and flow cytometry analysis. Cell lines and conditions are as described in section (A).
